# PSYCHOSOCIAL RESOURCES IN THE CONTEXT OF VULNERABILITY AMONG OLDER ADULTS: IMPLICATIONS FOR WELL-BEING

**DOI:** 10.1093/geroni/igad104.1912

**Published:** 2023-12-21

**Authors:** Aurora Sherman, Kira Birditt

## Abstract

Social ties and mastery may buffer detrimental effects of stressful life circumstances and vulnerability as individuals age. The purpose of this symposium is to understand the role of psychosocial resources for multiple dimensions of well-being across several age-related contexts. Luong et al. examined social ties’ effects on moving into senior housing using a burst design, finding that social ties were less helpful in the beginning of the move but regained beneficial effects on well-being later. Kinkade and Fuller examined the role of social integration on the longitudinal effects of the widowhood transition for well-being, concluding that increased social integration had protective impacts on depression and functional health among widows compared to non-widows. Birditt et al. assessed the implications of positive activities and social interactions among dementia caregivers using ecological momentary assessments. Positive activities and positive interactions with care recipients were associated with lower levels of daily burden and greater positive affect especially among caregivers who reported high levels of burden. Ryan examined spouses’ psychosocial characteristics on partners’ well-being and whether they varied by cognitive status of the partner. She found that spouses’ positive support from children amidst other negative psychosocial experiences was protective for their partner’s life satisfaction. Sherman found that self-esteem for mid-life and older women was associated with higher mastery and lower constraints beliefs and that both were mediated by frequency of self-objectification. Together, these papers highlight the importance of psychosocial resources for well-being as individuals as they age and face new or on-going challenges.

